# Antibacterial and Photocatalytic Properties of ZnO Nanoparticles Obtained from Chemical versus *Saponaria officinalis* Extract-Mediated Synthesis

**DOI:** 10.3390/molecules26072072

**Published:** 2021-04-04

**Authors:** Maria Antonia Tănase, Maria Marinescu, Petruta Oancea, Adina Răducan, Catalin Ionut Mihaescu, Elvira Alexandrescu, Cristina Lavinia Nistor, Luiza-Izabela Jinga, Lia Mara Diţu, Cristian Petcu, Ludmila Otilia Cinteza

**Affiliations:** 1 Physical Chemistry Department, University of Bucharest, 030018 Bucharest, Romania; maria.a.tanase@gmail.com (M.A.T.); petrutaoancea73@yahoo.com (P.O.); adina.raducan@g.unibuc.ro (A.R.); 2 Organic Chemistry Department, University of Bucharest, 030018 Bucharest, Romania; maria.marinescu@chimie.unibuc.ro; 3 Polymer Department, National Institute for Research and Development in Chemistry and Petrochemistry-ICECHIM, 202 Spl. Independentei, 060021 Bucharest, Romania; mihaescu_catalin96@yahoo.com (C.I.M.); elviraalexandrescu@yahoo.com (E.A.); lc_nistor@yahoo.com (C.L.N.); 4 Plasma and Radiation Physics, National Institute for Lasers, Atomistilor Str. 409, 077125 Bucharest-Magurele, Romania; izabela.jinga@inflpr.ro; 5 Microbiology Department, Faculty of Biology, University of Bucharest, 60101 Bucharest, Romania; lia-mara.ditu@bio.unibuc.ro

**Keywords:** ZnO nanoparticles, microwave synthesis, green synthesis, biocompatible photocatalysts

## Abstract

In the present work, the properties of ZnO nanoparticles obtained using an eco-friendly synthesis (biomediated methods in microwave irradiation) were studied. *Saponaria officinalis* extracts were used as both reducing and capping agents in the green nanochemistry synthesis of ZnO. Inorganic zinc oxide nanopowders were successfully prepared by a modified hydrothermal method and plant extract-mediated method. The influence of microwave irradiation was studied in both cases. The size, composition, crystallinity and morphology of inorganic nanoparticles (NPs) were investigated using dynamic light scattering (DLS), powder X-ray diffraction (XRD), SEM-EDX microscopy. Tunings of the nanochemistry reaction conditions (Zn precursor, structuring agent), ZnO NPs with various shapes were obtained, from quasi-spherical to flower-like. The optical properties and photocatalytic activity (degradation of methylene blue as model compound) were also investigated. ZnO nanopowders’ antibacterial activity was tested against Gram-positive and Gram-negative bacterial strains to evidence the influence of the vegetal extract-mediated synthesis on the biological activity.

## 1. Introduction

In recent years zinc oxide nanoparticles (ZnO NPs) have gained more interest in the scientific community due to their desirable properties and applications in different areas. Its properties include radiation absorption, thermodynamical stability, high photostability, high electrochemical coupling coefficient, low toxicity, biocompatibility, and biodegradability [1]. These provide a broad range of applications in different fields, such as UV-vis light-emitting devices, photocatalysts, sensors [2], pharmaceutical and cosmetic industries or medicine [3].

Zinc oxide nanoparticles vary in shapes and sizes due to multiple types of syntheses described in the literature. Certain parameters, such as temperature, pH or reaction time, can greatly impact ZnO NP morphologies. Therefore, selecting an appropriate synthesis procedure is an important factor. Some synthesis methods include hydrolysis in polyol media, chemical precipitation, template method, solvothermal and hydrothermal methods, microwave heating, thermal oxidation processes, sol–gel, biosynthesis, electrochemical deposition, mechanical milling, sonochemical routes and laser ablation [4]. The newly microwave-assisted approach exhibits rapid and sustainable processes with superlative benefits [5]. It has been extensively utilized to synthesize colloidal inorganic nanocrystals, including single metal nanocrystals, transition-metal oxides, and non-oxide semiconductors. One of the main limitations related to the classic synthesis and application of nano-ZnO is the problem of parameter repeatability. Most of the “wet methods” used for producing metal oxide nanoparticles generate powders with a broad particle size distribution, low crystallinity degree, variable morphology and low purity. Microwave hydrothermal synthesis of ZnO cuts all of the drawbacks mentioned above due to the fine control of the parameters. The power, heating frequency, and on/off irradiation cycles are the main heating parameters of a microwave oven, and each of them may have a great effect on the structure and properties of the final products [6]. Another major advantage over other methods is the reduction of reaction time. Thus, microwave synthesis may be 5–2500 times quicker than in the case of the same syntheses carried out by conventional thermal methods. Other advantages include instantaneous and precise electronic control of the reaction, fewer side reactions, rapid volumetric heating, higher yields of products, selectivity and purity of the obtained compounds, energy-saving, and a green chemistry approach. ZnO NPs synthesized with the use of the microwave hydrothermal method were characterized by numerous shapes described in the literature, such as belts-like, framework, candles, dandelion-like, disks, dumbbell-like, hollow structures, javelins, lamellar-like, petals, rhombic, sponge-like, tetrapod-like, tubes, whiskers, wires, flakes, plates, star-like, flower-like, needle-like, sheets, spherical particles and rods [7]. The correlation between the synthesis parameters and morphologies has been studied by numerous research groups. It was also reported that, by changing the microwave power, synthesis duration or pressure, the size and morphology of ZnO particles could be modified [6]. Another key factor, which affects the morphology of ZnO NPs is the pH and its influence over the geometry of complex compounds of zinc ions. Organic additives or auxiliaries are usually introduced to tune the shapes of the products effectively: metal sulfate hydrates for ZnO nanoplates or nanowires, citrate for oriented ZnO columns and plates, ascorbate for flower-like ZnO microstructures, and ethanolamine for nanorods [8].

There have been a large number of reports regarding the metal oxides (TiO_2_, WO_3_, ZnO, Nb_2_O_5_, and Fe_2_O_3_) in various processes of decontamination and disinfection [9]. Among these metal oxides, ZnO has a strong redox ability and non-toxic and controllable morphology, which are widely adopted in the field of photocatalysis as a suitable replacement for TiO_2_ nanoparticles. Zinc oxide is more common than TiO_2,_ and one of its advantages is that it can absorb a larger fraction of the solar spectrum than TiO_2_ [10]. ZnO as hexagonal nanostructured flowers, nanorods and nanowires showed higher photocatalytic properties than other morphologies [11]. Specific surface area controls the specified crystal face and has better photo-induced electron–hole diffusion, which leads to more active sites, easier mass transportation and a faster rate of reaction [12].

One of the main uses of ZnO as a photocatalytic agent is its effect on the photodegradation of water pollutants [13]. In addition, the photocatalytic performance of ZnO micro-/nanostructures was evaluated by the degradation of organic dyes under visible light irradiation [14,15].

Among various inorganic antimicrobial agents, zinc oxide (ZnO) nanoparticles have been verified with good antibacterial activity [16,17]. Moreover, ZnO has been widely used in food, cosmetics, and medicine for its safety and biocompatibility [18] since it is generally regarded as a safe material by the US Food and Drug Administration (21 CFR 182.8991). It is also well-known that ZnO NPs with a smaller size and larger concentration could achieve a better bactericidal response [19]. The high oxidative damage of reactive oxygen species could inhibit the growth of microorganisms. Due to its biocompatibility, safety, and activity against a broad spectrum of microorganisms, ZnO’s antibacterial activity has been well-investigated both in the presence (UV and visible) and absence of light. The mechanism of antibacterial activity of ZnO in the presence of light, studied by many researchers, is attributed to oxidative stress due to reactive oxygen species (ROS) [20], dissolution of zinc ions [21] and internalization of NPs leading to cell death [22]. Among these, ROS is considered to be the dominant mechanism. ROS production and antibacterial activity of ZnO are found to be higher under UV light than in visible light [23]. These ROS are aided by surface defects of ZnO, which are abundant in nanocrystalline form. It is also worth mentioning that the concentration and nanostructures morphology of ZnO may also influence the biologic activity [24,25,26]. Other cytotoxic effects of ZnO nanoparticles come from coordination effects of the metal ion with human proteins that can result in major structural changes [27,28] and also the disruption of homeostatic mechanisms [29]. The antibacterial efficiencies were affected by the physiological status of the bacterial cells, different morphologies and crystal growth habits, particle size and optical properties of ZnO samples.

For flower-like ZnO nanoparticles, the enhanced intensity of the photoluminescence band in the visible luminescence range resulted from the higher surface interstitial defects that reduce the electrons or holes recombination and consequently increase the antibacterial activities. ZnO flower-like nanoparticles have a large surface area and allow a more efficient contact with microbes than other particle morphologies. In addition, the enhancement of the antibacterial activity of synthesized ZnO can be attributed to the unique flower-like morphology, which can increase the surface–OH groups and the quantity of photo-generated electron–hole pairs available to participate in the photocatalytic reaction. ZnO flower-like nanoparticles were shown to exhibit significantly higher photocatalytic inactivation than ZnO rod- and sphere-like against *E. coli* and *S. aureus*. It was found that the antibacterial activity of ZnO increased with decreasing crystallite size [30]. Moreover, results indicated that multi-branched flower-like ZnO showed more remarkable, reliable, and stable antifungal activity compared with other ZnO nanoparticles [31].

In recent years, much attention has been paid to diminishing the major drawback of physical and chemical synthesis routes of ZnO nanoparticles, the high-energy consumption and the large use of toxic chemicals. Physical methods of ZnO fabrication, such as physical vapor, sputter or electric deposition, require both large amounts of energy and sophisticated equipment. On the other hand, the chemical methods developed, including sol–gel synthesis, co-precipitation, microemulsion-confined reaction, use various harmful reagents as capping agents (surfactants, thiols, amines) and also organic solvents in the case of solvothermal synthesis. The green synthesis approach implies the use of plants or microorganisms along with mild reaction conditions in the synthesis of nanoparticles [32]. Phytochemicals in plants act as reducing agents of metal salts and also as capping or stabilizing agents [33]. From a large amount of the literature on the biogenic synthesis of nanoparticles, very few report not only the characterization of the obtained NPs but also the composition of the vegetal extract. The role of the citrate, ascorbic acid, and other phytochemicals present in plant extracts was emphasized in the fabrication of Ag NPs mediated by vegetal extract [34]. In short, a Zn salt precursor and plant extract are mixed together under constant stirring to undergo a hydrolysis reaction in which Zn^2+^ ions turn to ZnO nanoparticles [35]. Depending on the Zn precursor, plants used for extracts and reaction conditions (usually time and temperature), one can obtain ZnO nanoparticles of various sizes and morphologies. ZnO nanoparticles with flower-like morphologies were obtained using *Kalopanax septemlobus*, *Astragalus membranaceus*, *Thlaspi arvense*, *Ruta chalepensis*, sea buckthorn fruit extract as reducing/capping agents and zinc acetate or zinc nitrate as salt precursors, in a one-pot synthesis conducted at temperatures varying from room temperature to 70 °C [36,37,38]. Other morphologies were obtained when using different combinations of plant extract and Zn salts, such as nanosheets using green tea leaves extract and Zn acetate [39], hexagonal nanoparticles using *Rhamnus Virgata* extract and zinc nitrate.

In most microwave-assisted green synthesis, the nanoparticles obtained have quasi-spherical morphology, and only a few have crystallinity studies [40]. Nanoparticles with good crystallinity are obtained when the pH is adjusted to alkaline with sodium hydroxide [41], and a calcination postsynthesis step is added to improve the performances of the obtained nanopowders [42].

*Saponaria officinalis L.*, also known as soapwort or fuller’s herb, is native to Europe and Asia and is cultivated throughout the world for its roots, which have found plenty of traditional uses [43]. Because the highest concentration of these compounds is found in the rhizomes, it is that part of the plant that is usually used in investigations. Its detergent properties have been well-known since ancient times, and its main traditional use has been soap. As an herbal medicine, it has been used as an expectorant in bronchitis and topically for skin complaints as well as rheumatic disorders.

Qualitative phytochemical screening of *S. officinalis* extracts revealed the presence of different phytochemical constituents like saponins, steroids, terpenoids, flavonoids, alkaloids, glycosides, phenols, tannins, carbohydrates and vitamin C [44]. Extracts from plants that contain saponins are rarely used in the green synthesis of metal oxide nanoparticles. To our knowledge, vegetal extract from *Saponaria Officinalis*, in particular, was extremely rare used, only two papers being published until now [45,46].

In this work, we report a microwave-assisted hydrothermal method to generate flower-like ZnO nanostructure and the influence of the presence of *Saponaria Officinalis* on the properties of the obtained nanopowders was investigated. The ZnO nanoparticles could form 3D flower-like nanostructures with the appearance of chrysanthemum and exhibit good antibacterial properties and photocatalytic activity.

## 2. Results and Discussion

### 2.1. Green Synthesis and Characterization of ZnO Nanoparticles

Reference ZnO nanoparticles were prepared using a simple precipitation method using an aqueous solution of zinc salt (nitrate) in an alkaline media, described by the following accepted reactions [47]:

Zn^2+^ + 2OH^−^ ↔ Zn(OH)_2_ (1)

Zn(OH)_2_ → Zn^2+^ + 2OH^−^ (2)

Zn^2+^ + 2OH^−^ ↔ ZnO + H_2_O (3)

Zn(OH)_2_ + 2OH^−^ ↔ [Zn(OH)_4_]^2−^ (4)

The critical parameters of this hydrothermal synthesis were temperature and Zn^2+^:OH^−^ ratio. Usually, in this hydrothermal synthesis, small quasi-spherical nanocrystals with various sizes are obtained due to the growth pattern governed by the effect of OH^−^ ions attachment at the interface of (0001) planes [48]. At low temperatures below 120 °C, ZnO rods are not obtained without the addition of structuring agents because there is not enough driving force to ensure the anisotropy of the shape.

**Figure 1:**
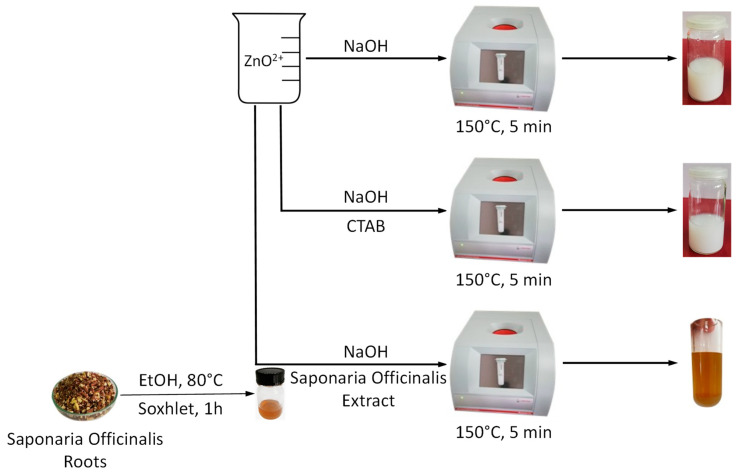
Schematic view of the microwave-assisted synthesis of ZnO NPs with natural and synthetic capping agents.

The classic hydrothermal synthesis requires a long period of time for the formation of nanoparticles with good crystallinity, from hours to days. Thus microwave irradiation was used in this study to reduce the time of the synthesis and to propose a green, energy-saving approach. The main routes of the fabrication of ZnO nanoparticles are schematically presented in Figure 1.

The effect of synthetic and natural surfactants (CTABr and saponins from *Saponaria officinalis* extract) as capping and structuring agents on the size and shape of the obtained ZnO nanopowders was investigated. The conditions of the synthesis and sample codification are presented in Table 1.

**Figure 2:**
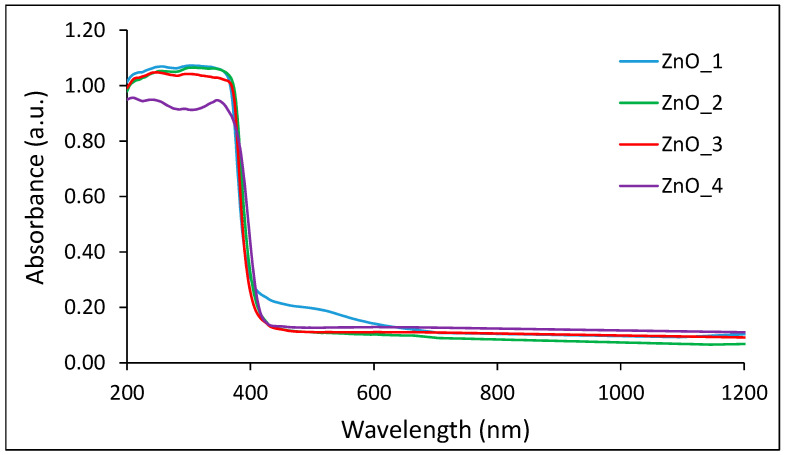
UV-vis spectra of ZnO nanopowders obtained in the microwave-assisted synthesis with various capping agents. ZnO_1 = sample synthesized in an alkaline medium without capping agent, ZnO_2 = synthesized in an alkaline medium in the presence of CTABr, ZnO_3 = synthesized in an alkaline medium in the presence of plant extract and ZnO_4 = synthesized only in the plant extract.

The ZnO formation was evidenced by the absorption spectra (Figure 2). The UV-vis diffuse reflectance spectra of all products show the strong absorption peak in the region below 400 nm, characteristic for the ZnO nanoparticles, similar to values reported in the literature [49].

The similar optical behavior of all samples, with close values of bandgap energy regardless of the differences in particle size, was reported previously for ZnO nanomaterials with dimensions larger than Debye length in ZnO [50].

**Figure 3:**
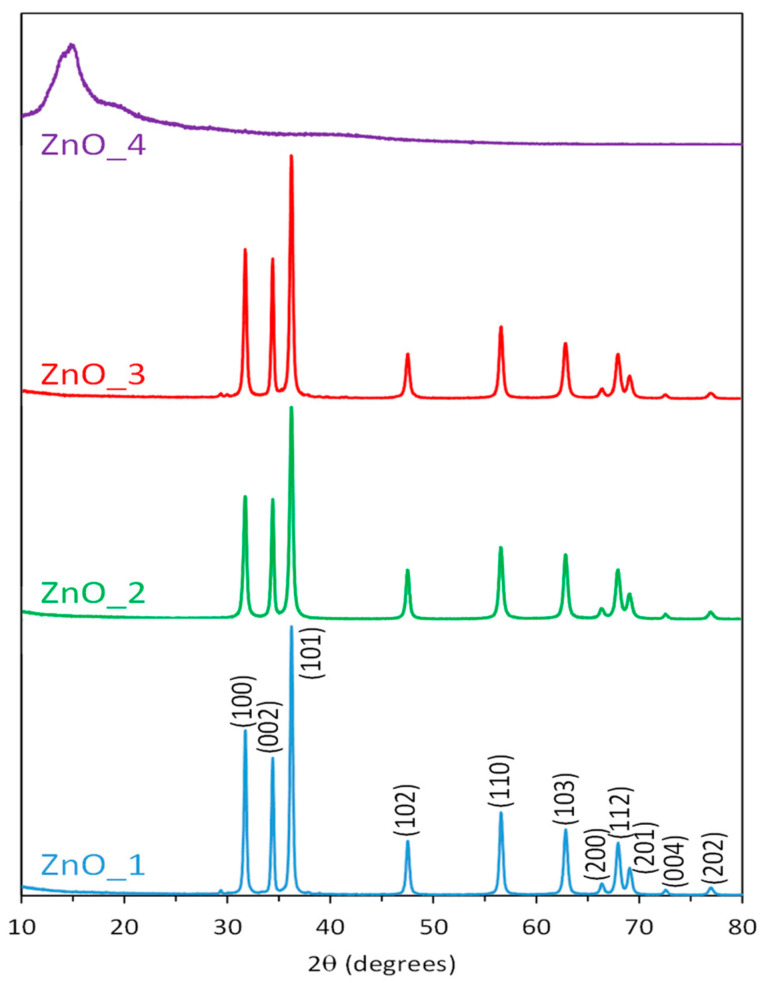
XRD diffractograms for the ZnO samples fabricated in microwave irradiation in the presence of cetyltrimethylammonium bromide (CTABr) and *S. officinalis* extract.

The composition and crystallinity of the samples were checked using XRD analysis, and the XRD pattern of the ZnO products obtained in various conditions of the synthesis (details of sample codification in Table 1) are shown in Figure 3.

For the sample prepared from zinc nitrate as a precursor in the presence of NaOH without structuring agent, and the ones with CTABr and plant extract as a capping and structuring agents, the same pattern was recorded, with peaks corresponding to the (100), (002), (101), (102), (210), (103), (200), (212) and (201) planes. This confirms the hexagonal wurtzite phase of the ZnO nanoparticles (Card No. 01–089-1397).

No additional peaks related to impurities were present in ZnO_1, ZnO_2 and ZnO_3 XRD spectra, which suggest the high purity of the products, while the sharpness of the signals confirmed the high crystallinity of the nanoparticles.

The sample prepared with zinc precursor in solely plant extract presence contained an amorphous phase. Thus the XRD spectrum had a noisy background with very low peaks. The only significant broad peak in the region of 10 degrees probably corresponded to the hydroxide nitrate hydrate (Zn_5_(OH)_8_(NO_3_)_2_(H_2_O)_2_) compound, which was reported to be obtained in the synthesis methods involving high concentrations of zinc nitrate in the reaction media [51].

The specific pattern of ZnO or Zn(OH)_2_ could not be observed, which suggests that in this condition, the transformation of the zinc precursor in ZnO material was very low.

**Figure 4:**
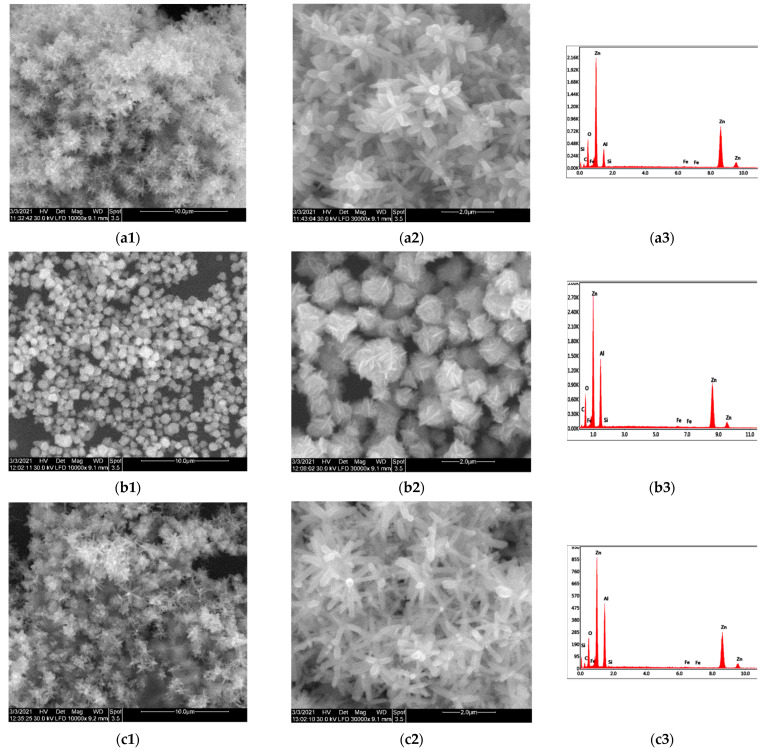

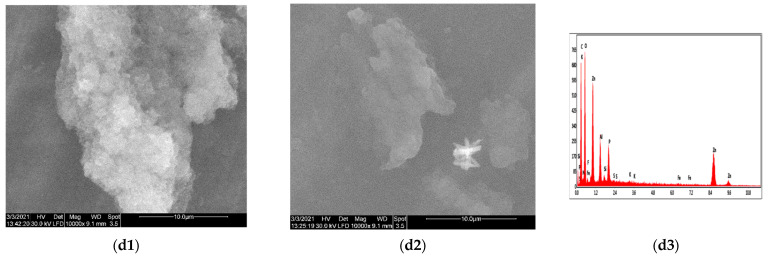
SEM micrographs, high-resolution images and EDX diagram of various flower-like 3D architectures of ZnO nanoparticles prepared in various conditions: (**a1**–**a3**) ZnO_1 sample prepared in an alkaline medium; (**b1**–**b3**) ZnO_2 sample prepared in an alkaline medium with CTABr; (**c1**–**c3**) ZnO_3 sample prepared in alkaline medium with *S. officinalis* extract; (**d1**–**d3**) ZnO_4 sample prepared only with *S. officinalis* extract.

The morphology of the prepared ZnO nanopowders was evaluated from the SEM micrographs. In Figure 4, the SEM images of the ZnO nanomaterials obtained in the microwave-assisted synthesis in the presence of various synthetic and natural structuring agents are presented.

In Figure 4a, the presence of a distinct 3D hierarchical structure of flower-like ZnO nanoparticles resembling chrysanthemum was observed in the product obtained from Zn nitrate in the presence of hydroxyl ions from NaOH. The formation of such structure, consisting of nanoneedles growth from a central point in different directions, was reported in the literature for the high ratio Zn^2+^/OH^−^ in a ZnO synthesis using KOH as OH^−^ source and heating treatment in an autoclave at 140 °C [52]. The role of the OH^−^ ion concentration in the growth mechanism of ZnO nanoparticles was related to the formation of the [Zn(OH)_4_] ^2−^ complexes. When the concentration was above their critical solubility, part of those complex ions turn in ZnO nuclei, while the other part exhibited preferential adsorption on the surface of the nuclei, promoting the growth rate along the c-axis direction and formation of anisotropic hexagonal prism-like particles [53].

In Figure 4a1, the details of the ZnO nanoparticles reveal a sword-like morphology of rods, with diameters 200–400 nm and length of 800–1000 nm. The nanorods form 3D complex architectures with a diameter ranging from 1000–2200 nm, which was consistent with the data obtained from DLS measurements.

The presence of synthetic surfactants in the reaction media was the most common way to promote the formation of 3D hierarchical structures, such as flower-like aggregated. Cetyltrimethylammonium bromide (CTABr), due to the amphiphilic chemical structure that allows micellization and surface adsorption, plays an interesting role in the fabrication of various ZnO nanocrystalline products. Depending on the reaction conditions in the presence of CTABr as a structuring agent, other various morphologies of ZnO nanopowders had been reported, from irregular to plates, bipyramids, cabbage-like or stars [54]. The concentration of the surfactant in the reaction media was considered to be one of the important factors that govern the growth of the initial ZnO nanoparticles, their shape evolution in rods or plated and the further formation of flower-like complex nanoarchitectures. The following reactions are proposed [55]:

CTABr → CTA^+^ + Br^−^ (5)

CTA^+^ + [Zn(OH)_4_] ^2−^ → CTA^+^−Zn(OH)_4_^2−^ (6)

CTA^+^ − Zn(OH)_4_^2−^ → ZnO + CTA^+^ + H_2_O + 2OH^−^ (7)

Higher concentrations of CTABr, such as 0.3 M, resulted in the destruction of aggregated; only plated or rods could be obtained.

In the synthesis proposed in the present work, the moderate concentration of CTABr was used, together with the effect of fast and concentrated heating process under microwave irradiation, and the morphology of the ZnO nanoparticles obtained was clearly well-defined 3D hierarchical rose structures. In Figure 4b1, the presence of the flower-like particles all over the sample could be seen. The higher magnification image (Figure 4b2) shows that the flower consisted of a large number of nanoplates that had a thickness of ∼50 nm, closely packed around a center.

The proposed mechanism that explains the CTABr role emphasizes that the ion pairs formed due to electrostatic interaction between CTA^+^ and Zn(OH)_4_^2−^ were responsible for the faster reaction rate, while the formation of micelles and lamellar liquid crystal phased at high concentrations of CTABr promote the oriented attachment of ZnO nuclei and further development of the ZnO nanoplates. The decrease in the surface energy produced the aggregation of the nanoplates in 3D hierarchical structures resembling rose flowers [55].

The complex composition of *S. officinalis* extract, with flavonoids, alkaloids, glycosides, phenols, tannins, carbohydrates, etc., could act as reagents in ZnO formation and capping agent in controlling the crystal growth, thus reducing the size of the nanoparticles. One could also expect that saponin surfactants to adsorb on the ZnO nuclei, similar to CTABr behavior, and flower-like 3D aggregates could be obtained.

In Figure 4c1, the morphology of the ZnO nanoparticles obtained when the synthetic surfactant CTABr was replaced by the natural one, the saponins containing plant extract, was presented. The formation of the flower-like aggregates could be observed, with structures formed by numerous hexagonal nanorods, without the pointed edge, with diameters of approximately 100–200 nm and length about 600–1000 nm. The structure of 3D aggregated was similar to the one formed in the reaction without surfactant, while the aspect of the nanorods was different, without the sharp edge like a sword, respectively.

From previously reported studies, the most abundant components of *Saponaria officinalis* extract were saponins, steroids, terpenoids, flavonoids, alkaloids, glycosides, phenols, tannins, carbohydrates. The root extract was considered to contained approximately 20% saponins, with various chemical structures, the four representative types being Pentacyclic Oleanane, Ursane and Lupane, as well as Tetracyclic one. All saponins in the *S. officinalis* were natural surfactants with good surface-active properties, but they do not exhibit the self-assembling properties, do not aggregate in micellar structures, such as synthetic surfactants do [44].

Thus, the presence of the surfactants from *S. officinalis* could not produce the modification of the shape of ZnO nanoparticles in plates. The effect is only as a capping agent since the diameter of rods was reduced, and the size of the 3D aggregate was also decreased compared to flower-like particles formed in the samples without surfactant and with CTABr.

The plant extract, despite its complex composition with phytochemicals containing many reactive groups, was not able to act as an efficient, reducing agent to produce the formation of a large number of crystalline ZnO nanoparticles. The product obtained in the absence of NaOH, with solely plant extract, under microwave irradiation shows an amorphous mass of agglomerated particles with small size, with irregular shape. Rarely, some star-like aggregated with size in the 1–2 µm range formed by the connection of nanorods could be observed (Figure 4d2).

The EDX spectra (Figure 4a3–d3) of the produced ZnO powders confirm for all the four samples the composition with Zn and O according to the main peaks in the graphs. Additional signals related to P, N, and high content of O in the spectra of the ZnO_4 sample prepared only in *S. officinalis* extract due to the presence of residual plant extract that remained trapped into the reaction product.

**Figure 5:**
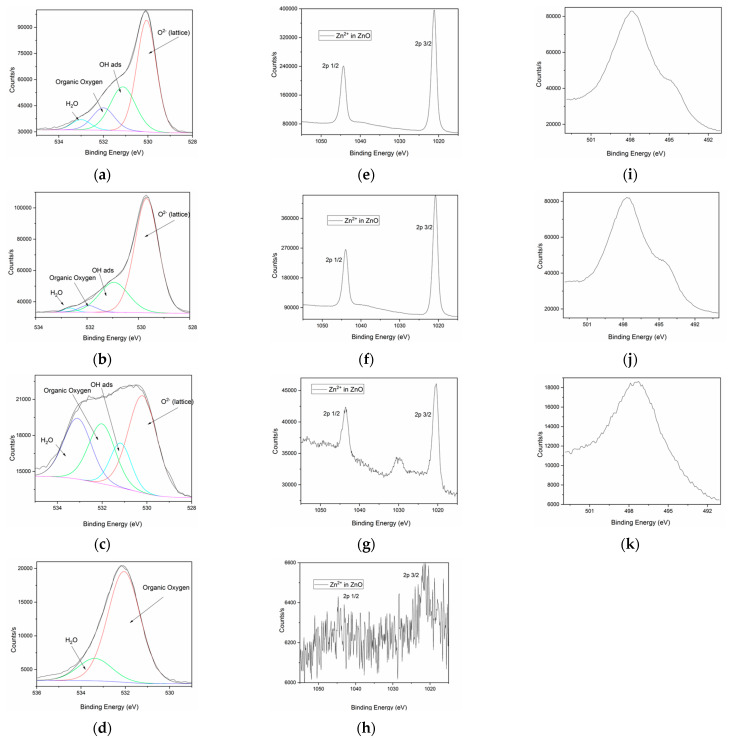
XPS high-resolution spectra in O1s region for ZnO samples obtained in various conditions (details in Table 1): (**a**) ZnO_1; (**b**) ZnO_2; (**c**) ZnO_3; (**d**) ZnO_4. XPS high-resolution spectra in Zn2p region for ZnO samples obtained in various conditions: (**e**) ZnO_1; (**f**) ZnO_2; (**g**) ZnO_3; (**h**) ZnO_4. XPS high-resolution spectra in ZnLM2 region for ZnO samples obtained in various conditions: (**i**) ZnO_1; (**j**) ZnO_2; (**k**) ZnO_3.

The composition of ZnO products obtained in various conditions of reaction was also investigated using XPS. Their spectra are presented in Figure 5.

In the deconvoluted high-resolution spectrum of the O1s singlet (Figure 5a–d), four oxygen chemical species in the BEs region 535–528 eV could be observed for the ZnO particles prepared in an alkaline medium with NaOH (ZnO_1, ZnO_2 and ZnO_3). Thus, O^2−^ bonded in the ZnO lattice was assigned for the O1s at 530.5 eV, the OH groups and water molecules adsorbed on the surface were detected at 531.8 eV and 533.1 eV, respectively. A small fraction of organic oxygen was attributed for O1s located at 532.2 eV. For sample ZnO_4, prepared only in the presence of plant extract, the high-resolution spectrum of O1s reveals only two oxygen species attributed to organic oxygen located at 532.2 eV and water molecules adsorbed on the surface were detected at 533 eV. Zinc 2p high-resolution spectra (Figure 5e–h) reveals the presence of Zn^2+^ oxidation state typical for ZnO at the binding energies (BEs) Zn 2p 3/2 = 1021.5 eV and 1044.3 eV for Zn2p 1/2. Both signals were present in the spectra recorded for samples ZnO_1, ZnO_2, and ZnO_3 synthesized using NaOH as a reducing agent, without capping agent or in the presence of CTABr or plant extract. For the sample ZnO_4, prepared only with *S. officinalis* extract, the spectrum was very noisy, and no distinct signal could be evidenced due to the very small content of ZnO nanoparticles formed in this sample. Zn LM2 high-resolution spectrum confirmed the presence of Zn^2+^ oxidation state-specific for ZnO at 498 eV.

Further analysis of the size and surface potential of the ZnO products obtained in various conditions was performed by dynamic light scattering (Table 2). The results of aggregate dimensions were consistent with the information from SEM micrographs discussed in the above section.

The ZnO flower-like particles obtained from the Zn nitrate hydrate/NaOH mixture showed an average size of 3D architectures of 1244 nm as measured as intensity mode, with a rather high polydispersity index (PdI) of 0.363, due to the presence of an additional population of large aggregated in the range of 5500 nm.

As expected, the presence of the surfactant CTABr acting as a capping and stabilizing agent reduced the dimension of the ZnO flower-like structures. The average value of the size was 1068 nm, and the sample exhibit a monomodal distribution and high monodispersity (the PdI value of 0.218). The ZnO nanopowder ZnO_3 prepared in the presence of NaOH and *S. officinalis* extract possesses the smallest 3D flower-like architectures, with an average diameter of 863 nm and monomodal distribution. The sample was rather monodispersed with a PdI of 0.285. The ZnO_4 sample, prepared only with the plant extract addition, showed the presence of small particles around 40 nm as a major population and another population of higher particles of 244 nm, with a high polydispersity (PdI value of 0.358). The small grains tend to form larger aggregated, which corresponded to the aspect of the sample observed in the SEM images.

Nitrogen adsorption−desorption measurements were performed to investigate the textural properties of ZnO particles by estimating the surface area and total pore volume after sample calcination at 600 °C, according to the procedure described above. The resulted for the samples ZnO_1 prepared in an alkaline media without capping agent, ZnO_2 with CTABr and ZnO_3 with plant extract as capping agents are summarized in Table 3. Sample ZnO_4, prepared only with the aid of plant extract, was not subjected to the analysis since the SEM images and XPS spectra suggest that a very small amount of ZnO nanoparticles was obtained in this case.

For the analyzed samples, all textural parameters were much lower than those usually recorded for mesoporous samples. Thus, the BET surface area of ZnO_1 and ZnO_3 samples, both with chrysanthemum-like morphology of 3D aggregated, was determined to be 2.292 m^2^/g and 3.257 m^2^/g with 0.0116 cm^3^/g and 0.0094 cm^3^/g total pore volume. The obtained results indicate the presence of very few pores, probably formed by sintering of the inorganic ZnO network during the calcination process. A small number of slit-like pores could form in this way. The difference in the BET surface was mainly due to the sizes of ZnO crystallites and of the flower-like architectures. However, for sample ZnO_2 (with a rose-like morphology) was recorded a higher specific surface area (8355 m^2^/g) compared to samples ZnO_1 and ZnO_2, but a smaller value for total pore volume, 0.0036 cm^3^/g, respectively. The different sizes and morphology of the ZnO nanosheets that form the rose-like aggregated were responsible for the modification of textural properties.

Although the specific surfaces of the studied ZnO particles were high at the macro-level (see SEM images), both samples show very low surface areas, which could be generated by the presence of mesopores. Considering the crystalline nature of the ZnO flower-like consisting of hexagonal prism nanorods, the measured values of BET-specific surfaces and total pore volumes were in the expected range.

**Figure 6:**
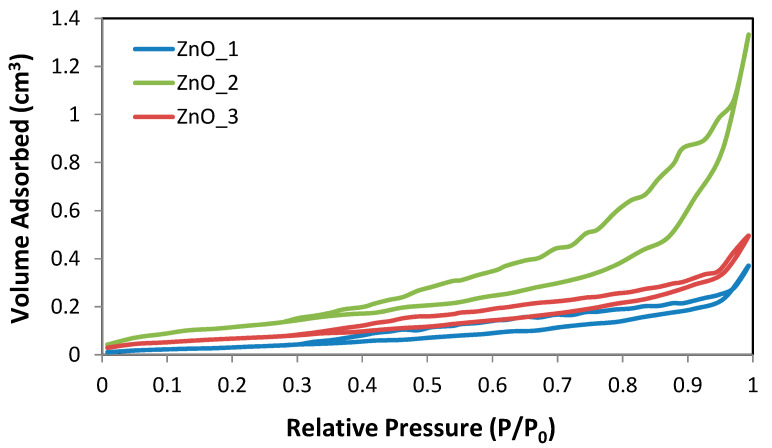
Nitrogen adsorption–desorption isotherms of the calcinated ZnO_1, ZnO_2 and ZnO_3 samples.

In Figure 6, the BET isotherms of the calcinated ZnO samples are presented.

Figure 6 reveals type IV physisorption isotherms and very small H3 hysteresis loops for all samples and very low nitrogen uptake at high relative pressure. This very low physisorption in ZnO particles confirmed the crystalline nature of both samples.

**Figure 7:**
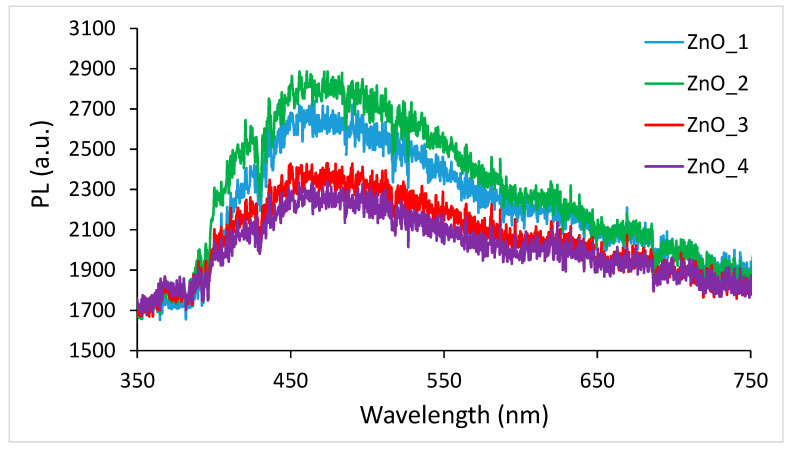
The photoluminescence (PL) spectra of ZnO nanopowders. ZnO_1 = sample synthesized in an alkaline medium without capping agent, ZnO_2 = synthesized in an alkaline medium in the presence of CTABr, ZnO_3 = synthesized in an alkaline medium in the presence of plant extract and ZnO_4 = synthesized only in the plant extract.

The photoluminescence of the synthesized ZnO NPs was measured at room temperature, and the spectra are shown in Figure 7. At the excitation, with a 365 nm source, a broad emission peak was recorded for all prepared samples at 460 nm corresponding to ZnO nanoparticles.

No distinct signals were present in the visible region 500–600 nm, usually emission related to surface defects. However, the spectra were very broad due to the transition between the single-charged oxygen vacancies [56].

### 2.2. Photocatalytic Activity

**Figure 8:**
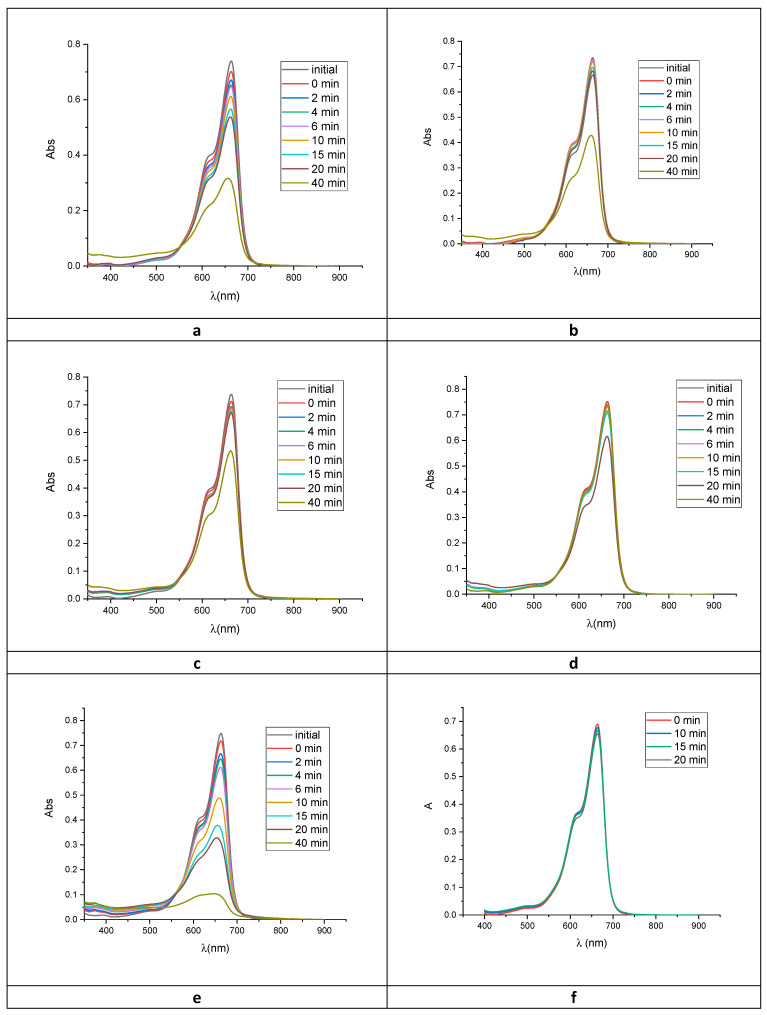
UV-vis spectra of methylene blue (MB) photodegradation under visible light (1.5 mg catalyst, 5 ppm MB) (**a**) ZnO_1, (**b**) ZnO_2, (**c**) ZnO_3, (**d**) ZnO_4, (**e**) ZnO (commercial 100 nm), (**f**) photolysis (without ZnO).

The photocatalytic activity of the phytosynthesized ZnO was evaluated by the degradation of methylene blue (MB) as a model organic pollutant under visible light. For irradiation, a 250 W medium-pressure Hg irradiation lamp was used, with emission in the 404.5–407.8, 435.8, 546.1 and 570–577 nm range. For the photodegradation, 1.5 mg ZnO was added into a beaker containing 15 mL of MB dye solution (5 mg/L). MB solution with the appropriate amount of catalyst was stirred for 30 min in the dark to achieve the adsorption equilibrium of MB onto the semiconductor surface. Degradation of MB was monitored by a recording of Vis spectra at several irradiation times. MB displays blue color in water and absorbs in the visible region at 612 and 664 nm. Figure 8 shows the time-dependent vis spectra of MB under visible radiation with and without ZnO NPs photocatalyst. Furthermore, a blank experiment of MB photolysis under the same conditions was done, showing that the photolysis was almost negligible as compared with the photocatalytic degradation.

The degradation efficiency was calculated from Equation (9) described in Section 3.4., after 40 min of irradiation (*t*).

**Figure 9:**
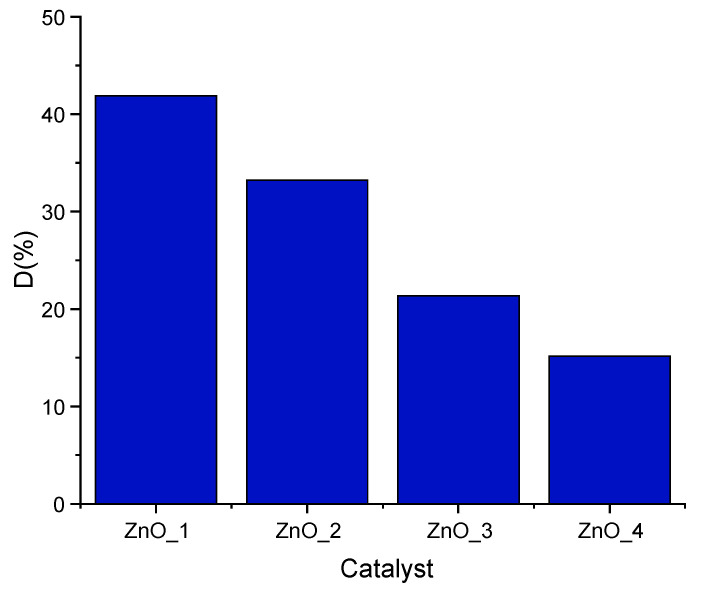
Degradation efficiencies of ZnO samples for MB photodegradation after 40 min (1.5 mg ZnO and 5 ppm MB).

As compared with the reference, where the degradation efficiency is 75%, all other samples showed a lower activity as photocatalysts (Figure 9).

When exposed to visible light, ZnO_1 samples exhibited the highest activity among the synthesized samples. About 42% of MB was degraded after 40 min irradiation by ZnO_1. The other samples showed to be less active, with degradation degrees of 33% for ZnO_2, 21% for ZnO_3 and only 15% for ZnO_4. The value obtained for ZnO_4 was slightly greater than those obtained for simple photolysis of MB, 13%.

Since it was reported that the photocatalytic degradation of MB by ZnO nanoparticles follows a pseudo-first-order kinetic, from the kinetic curves *A = f(t)*, the pseudo-first-order constants were estimated from the linear regression of the equation:

(8) $$ln\left(\frac{A_{0}}{A_{t}}\right)=k_{app}t$$

**Figure 10:**
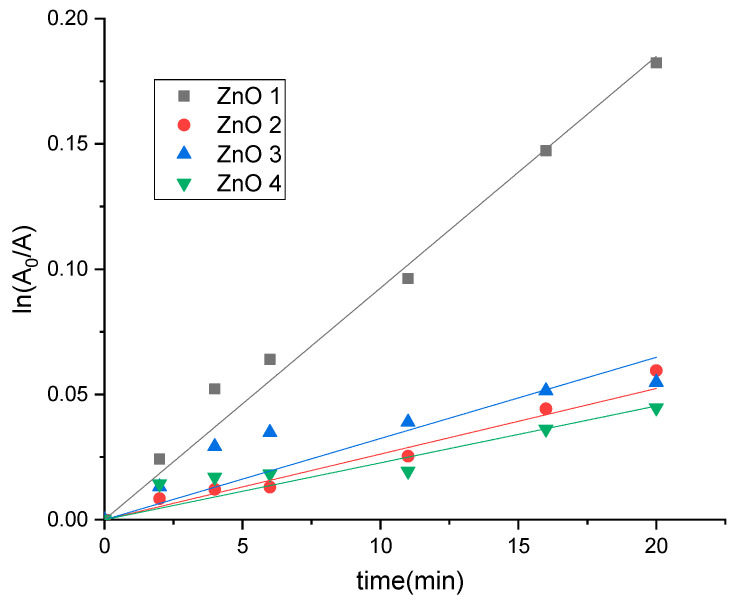
First-order kinetics plot of ln(*A_0_*/*A*) versus irradiation time for photocatalytic degradation of methylene blue under visible over different ZnO synthesized samples.

The results are presented in Figure 10.

For all ZnO samples, the reaction followed apparent first-order kinetics. The values of pseudo-first-order rate constants decrease in the following order: ZnO_1, ZnO_2, ZnO_3 and ZnO_4.

The half-life time of the reaction was one of the most useful parameters to evaluate the behavior of catalysts; the half-life time could be calculated from the pseudo-first-order constant *k_app_* experimentally determined from Equation (10) described in Section 3.4.

**Figure 11:**
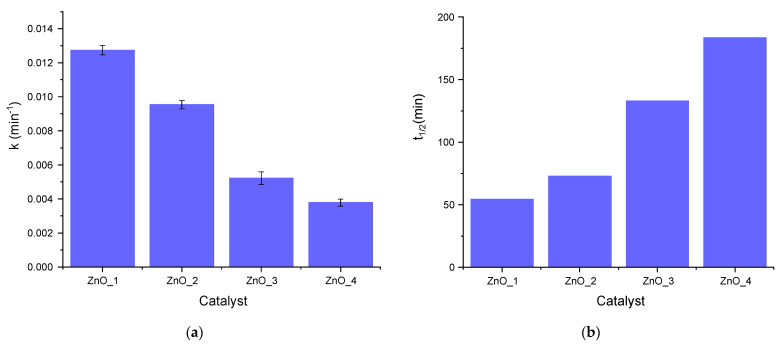
Pseudo-first-order rate constants (**a**) and half-life times (**b**) for MB photodegradation in the presence of synthesized ZnO samples (1.5 mg ZnO and 5 ppm MB).

The rate constants were smaller for all catalysts as compared with the reference ZnO (k = 0.0344 min^−1^ and *t*_1/2_ = 20 min). The values of rate constants and corresponding half-life times for ZnO samples are presented in Figure 11.

From the values of pseudo-first-order rate constants, it can be observed that sample ZnO_1 was the most active, having the smallest half-life time (around 55 min) compared with the other synthesized ZnOs. Samples ZnO_2 and ZnO_3 were less active, while sample ZnO_4 shows no catalytic efficiency on MB photodegradation, the rate constant being equal to the one obtained for simple photolysis of MB. For the sample ZnO_4, prepared only with *S. officinalis* extract, the results are consistent with the presence of a very small amount of ZnO nanoparticles in the plant extract matrix. The half-life time of sample ZnO_3 with *S. officinalis* extract was unexpectedly high; even the size of the nanoflowers was smaller compared to samples ZnO_1 without capping agent and ZnO_2 with CTABr.

However, these data show that samples ZnO_3 obtained from green synthesis with *S. officinalis* extract as a capping agent could be used as catalysts for MB degradation under visible light, with lower efficiency than the nanoparticles capped with the synthetic surfactant, but with better biocompatibility and low toxicity for the environment.

### 2.3. Antibacterial Activity

**Figure 12:**
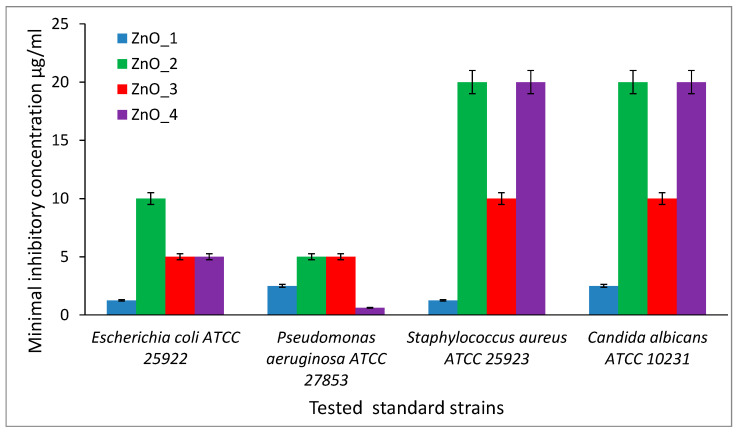
Minimal inhibitory concentration values (µg/mL) for the ZnO nanoparticles prepared with various capping agents against tested strains. ZnO_1 = sample synthesized in an alkaline medium without capping agent, ZnO_2 = synthesized in an alkaline medium in the presence of CTABr, ZnO_3 = synthesized in an alkaline medium in the presence of plant extract and ZnO_4 = synthesized only in the plant extract.

The quantitative results showed the antibacterial efficiency of tested ZnO nanopowders toward Gram-negative bacteria represented by *E. coli* ATCC 25922 and *P. aeruginosa* ATCC 27853 strains, compared with Gram-positive bacteria represented by *S. aureus* ATCC 25923 strain and yeast strain *C. albicans* ATCC 10231 (that also exhibit Gram-positive characteristic), (Figure 12). *S. officinalis* extract was tested in the same conditions, and for all four microbial strains, the inhibition of growth was achieved at 1/10 dilution of the original vegetal extract used in the synthesis of ZnO nanoparticles.

For *E. coli* ATCC 25922, the MIC values were in the range of 1.25 µg/mL and 10 µg/mL, with the lowest value for ZnO_1 sample (1.25 µg/mL), while *P. aeruginosa* ATCC 27853 strain expressed MIC values between 0.625 µg/mL and 5 µg/mL, with the lowest value for ZnO_4 nanopowder (0.625 µg/mL).

For *S. aureus* ATCC 25923, the MIC values were in the range of 1.25 µg/mL and 20 µg/mL, and for *C. albicans* ATCC 10230, the MIC values were between 2.5 µg/mL and 20 µg/mL. For both strains, the most active sample was the ZnO_1 containing chrysanthemum nanoflowers without a capping agent, expressing the lowest MIC value. The sample ZnO_2 prepared with CTABr as a capping and structuring agent had the highest MIC values, 10 µg/mL for *E. coli*, 5 µg/mL for *P. aeruginosa* and 20 µg/mL for *S. aureus* and *C. albicans*, respectively. Thus, at a similar size, the rose aspect of the flower-like aggregate proved to be less effective in the inhibition of the microbial growth, despite the presence of some traces of CTABr adsorbed on the ZnO nanoparticle surface.

The antimicrobial efficiency of the ZnO_3 sample containing nanoflowers prepared with *S. officinalis* extract was intermediate between the performances of nude ZnO chrysanthemum nanoflowers, and the ones of CTABr capped nanoflowers. The MIC values were 5 µg/mL for *E. coli* and for *P. aeruginosa* and 10 µg/mL for *S. aureus* and *C. albicans*, respectively.

The MIC values recorded from the product obtained using only *S. officinalis* extract act reagent was due mainly to the presence of the solid particles of extract and could not be related to the ZnO nanoparticles in the sample.

## 3. Materials and Methods

### 3.1. Materials

Zinc nitrate-6-hydrate (Zn(NO_3_)_2_·6H_2_O) (purity 98%), sodium hydroxide (NaOH) (pellets, purity ≥98%), and cetyltrimethylammonium bromide (CTABr) (powder, purity ≥99%) were all Aldrich reagents purchased from Sigma-Aldrich (Merck Group, Darmstadt, Germany) and used as received, without any additional purification. Ethanol 96% was purchased from ChimReactiv SRL (Bucharest, Romania). Bidistilled water was produced using a laboratory ultrapure water purification system (Milli-Q^®^ Advantage A10, Merck Millipore, Germany).

#### Plant Extract

The extraction procedure from the roots of the *S. officinalis* plant was performed according to a method developed in our laboratory, based on previous studies on the extraction method from Saponaria species to optimize the time required to achieve the dissolution of the active substances [43]. *Saponaria officinalis L.* rhizomes were bought from the local market, from SC Xtreme ANG Marketing SRL (Bucharest, Romania). Shredded roots of *Saponaria officinalis L.* underwent extraction in a Soxhlet apparatus for 1 h at 80 °C using 10 mL of solvent (ethanol–water in the mass ratio 1:1) per gram of vegetal material. The plant extract obtained was further filtered through a syringe filter Minisart^®^ 0.8 µm (Sartorius, Gottingen, Germany) and stored in brown glass vials at refrigerator until was used in experiments, no more than a week.

### 3.2. ZnO Nanoparticles Synthesis

Zinc oxide nanoflowers were fabricated via microwave heating from an aqueous solution of zinc nitrate-6-hydrate (Zn(NO_3_)_2_·6H_2_O) and different co-reactants through 5 different procedures. For the synthesis of sample ZnO_1, 1.2 g of Zn(NO_3_)_2_·6H_2_O were dissolved in 32 mL of deionized water (S_1_) under vigorous stirring. Then, a solution of 1.6 g of sodium hydroxide (NaOH) dissolved in 16 mL of deionized water was added (S_2_). The mixture was stirred for 5 min at room temperature until a milky aspect was obtained. Subsequently, the suspension was added to the microwave reactor Monowave 200 (Anton Paar GmbH, Graz, Austria). The conditions for preparing sample ZnO_2 were the same as for specimen ZnO_1, except that a solution, which consisted of 2.4 g of cetyltrimethylammonium bromide (CTABr) dissolved into 32 mL of deionized water (S_3_) was added over the first two solutions (S_1_ + S_2_). For the synthesis of ZnO ZnO_3, 16 mL of alcoholic extract of *Saponaria Officinalis* and 16 mL of deionized water were added over S_1_ + S_2_. Sample ZnO_4 was prepared by adding 16 mL of alcoholic extract of *Saponaria officinalis* and 16 mL of deionized water over S_1._ Each of the obtained mixtures was transferred into a microwave vial, rapidly heated at 150 °C and maintained at this temperature for 5 min without any stirring. After cooling, the obtained ZnO nanostructures were washed with deionized water several times and dried at 90 °C for 2 h to form powder products.

### 3.3. ZnO Nanoparticles Characterization

Crystalline phases identification was achieved by X-ray diffraction (XRD) with an X’Pert Pro MPD diffractometer from Panalytical using Cu Kα radiation (λ = 1.5406 Å) set to work in Bragg–Brentano geometry with 2θ = (20–80)°, a speed of 2 sec/step and 0.02° step, whereas in the extract analysis 2θ = (0–80)°. Diffuse reflectance electronic spectra were recorded at room temperature with a Jasco UV-vis-NIR V670 spectrometer (Jasco, Easton, MD, USA) in the 200–1500 nm range, using Spectralon as reference.

Photoluminescence spectra were recorded on the dry samples, on a modular device with OceanInsight XDH spectrometer coupled to a LED source with 365 nm emission and optical fiber in reflection mode (OceanInsight, FL, USA).

Dynamic light scattering (DLS) and laser Doppler velocimetry (LDV) techniques were used to determine the average particle size, particle size distribution and, respectively, Zeta potential (Zetasizer NanoZS instrument Malvern Instruments Ltd., Malvern, UK). Before measurements, the samples were dispersed in distilled water, using a concentration of 1 mg/5 mL and then ultrasonicated for ~3 min in an ultrasonic bath.

ZnO particle’s morphology was investigated through scanning electron microscopy (SEM) images (FEI Quanta 200, Eindhoven, The Netherlands), without covering the samples and using a large field detector (LFD), high vacuum (HV) working mode and 30 kV accelerating voltage. The specimens for SEM investigations were cast on aluminum stubs, using the same aqueous dispersions that were prepared for the DLS and LDV measurements (1 mg/5 mL). For analyzing the elemental composition and structure of the synthesized ZnO samples, an energy-dispersive X-ray microanalysis system (EDX) (EDAX, AMETEK Materials Analysis Division. AMETEK, Inc, Berwyn, PA, USA) was used.

The nitrogen adsorption–desorption isotherms were recorded at 196 °C, using a NOVA 2200e automated gas sorption instrument (Quantachrome Instruments, Hartley Wintney, UK). Prior to measurements, samples were calcinated using the following heating program: 10 min from 30 °C to 100 °C; 30 min at 100 °C; 30 min from 100 °C to 250 °C; 30 min at 250 °C; 1 h from 250 °C to 500 °C; 2 h at 500 °C; 30 min from 500 °C to 650 °C; 1 h at 650 °C; cooling to room temperature and then were degassed under vacuum at 300 °C for ~16 h. The Brunauer–Emmett–Teller-specific surface areas of samples were calculated from adsorption data at a relative pressure range of 0.08–0.3. The total pore volumes were evaluated from the adsorbed amount of nitrogen at a relative pressure of 0.986.

The chemical composition of the samples was determined by XPS. All measurements were performed with ESCALAB Xi+ (Thermo SCIENTIFIC Surface Analysis) equipped with a multichannel hemispherical electron analyzer (dual X-ray source) working with Al Kα radiation (hν = 1486.2 eV).

As energy reference was used C 1 s, which has 284.8 eV. XPS data were recorded on slightly pressed power materials that had been outgassed in the pre-chamber of the setup at room temperature at a pressure of <2 × 10^−8^ Torr to remove the chemisorbed water from their surfaces. The surface chemical compositions and oxidation states were estimated from the XPS spectra by calculating the integral of each peak after subtraction of the “S-shaped” Shirley-type background using the appropriate experimental sensitivity factors using Avantage software (version 5.978).

The XPS spectrum was analyzed using the NIST X-ray photoelectron spectroscopy database.

### 3.4. Photocatalytic Properties Assessment

The photocatalytic activity of the ZnO nanoparticles was tested by the photodegradation of methylene blue (MB) in an aqueous solution under visible light radiation. For irradiation, a 250 W medium-pressure Hg irradiation lamp was used, with emission in the 404.5–407.8, 435.8, 546.1 and 570–577 nm range.

The experimental design was optimized using commercially available ZnO spherical nanoparticles, according to a procedure developed in our laboratory. MB stock solution (200 ppm) was prepared in bidistilled water. For investigation of the initial catalyst loading on the degradation, the concentration of MB in the reactor was 4 ppm and the catalyst in the range 0–300 ppm, while for investigation of initial dye concentration on the degradation, the concentration of MB was ranging between 1.7 and 5.3 ppm with a constant catalyst concentration of 100 ppm.

MB solution with the appropriate amount of catalyst was stirred for 30 min in the dark to achieve the adsorption equilibrium of MB onto the semiconductor surface. The photocatalytic activity of ZnO particles for the degradation of MB was examined using Vis spectroscopy. MB displays blue color in water and absorbs in the visible region at 612 and 664 nm.

The degradation efficiency was calculated as:

(9) $$D\left(\%\right)=\frac{A_{0}-A_{t}}{A_{0}}\;100$$

where $A_{0}$ is the absorbance of the solution at 664 nm before the irradiation, and $A_{t}$ the absorbance at a certain time of irradiation (*t*).

The half-life time of the reaction is one of the most useful parameters to evaluate the reaction rate for first-order kinetics; the half-life time can be calculated from the pseudo-first-order constant *k_app_* experimentally determined as follows:

(10) $$t_{1/2}=\frac{ln2}{k_{app}}$$

The half-life times showed a significant decrease from almost 300 min (5 h) in the absence of ZnO to 4 min in the presence of 300 ppm ZnO. In the case of MB variation, the half-life times decrease from 27 min to 11 min when the initial concentration of MB increases from 1.7 ppm to 5.3 ppm.

For the optimum conditions obtained for ZnO, the synthesized nanoparticles were tested as photocatalysts for MB degradation. The initial concentration of dye was 4 ppm MB, and the loading of catalyst was 100 ppm. The solutions were irradiated for a minimum of 40 min until the absorbance of MB decreased up to 75%.

### 3.5. Antibacterial Activity Assessment

For the antimicrobial assays, the following standard microbial strains were used: *Staphylococcus aureus* ATCC 25923, *Pseudomonas aeruginosa* ATCC 27853 and *Pseudomonas aeruginosa* 5399 clinical strain, *Escherichia coli* ATCC 25922 and *Candida albicans* ATCC 10231. To perform the experiment, two successive passages were made on an appropriate nutrient-agar medium, followed by incubation at 37 °C, for 24 h. All the microbial strains are included in the microbial collection of the University of Bucharest, Faculty of Biology, Microbiology Department.

The qualitative screening of the antimicrobial properties was performed by an adapted spot diffusion method, according to Clinical Laboratory Standard Institute (CLSI 2021, Annapolis Junction, MD, USA) standards, to evaluate the inhibitory efficiency of tested compounds. The microbial inocula were prepared from 18 h cultures and adjusted to a density of 1.5 × 10^8^ CFU/mL, using the 0.5 McFarland standard. Petri dishes with Mueller–Hinton agar (MHA), respectively Sabouraud agar (SDA), were seeded with microbial inocula. An amount of 10 µL solution of each sample was spotted on the sterile paper disc with 6 mm diameters, previously arranged on the medium surface. The plates were left at room temperature to ensure the equal diffusion of the compound in the medium and then incubated at 37 °C for 24 h. The antimicrobial activity was evaluated by measuring the diameters of the inhibition zones.

For MIC (minimum inhibitory concentration) testing, a broth microdilution method for the prepared working solutions was performed, according to with CLSI procedure. Binary dilutions of each tested compound were performed in broth medium, starting with 20 µg/mL concentration calculated for ZnO NPs, in a range between 20 µg/mL and 0.0390625 µg/mL. Further, 15 μL of microbial suspension adjusted to 1.5 × 10^7^ CFU/mL were added to each well. The MIC values were spectrophotometrically established (absorbance reading at 600 nm using BioTek Synergy -HTX ELISA multimode reader). Each experiment was performed in triplicate and repeated on at least three separate occasions.

For biological tests, significant differences between the means of triplicate experiments and the control were determined by using one-way ANOVA statistical analysis. All data are presented as mean values ± the standard deviations (SD).

## 4. Conclusions

A simple microwave-assisted hydrothermal synthesis of ZnO nanopowders was studied to investigate the possibility of designing a green alternative to solvothermal or hydrothermal long-term, energy-consuming autoclave methods. The influence of the phytochemicals from *Saponaria officinalis* extract compared to CTABr as structuring, and capping agents were also investigated. The plant extract could serve as a capping and structuring agent but is not efficient in transforming zinc precursor in ZnO nanoparticles at an acceptable rate without further thermal processing. The ZnO nanopowders were obtained as 3D hierarchical architectures with morphologies like a chrysanthemum or rose aspect. The crystallinity, composition and size of the nanoparticles were investigated by XRD, UV-vis and DLS. The photocatalytic effect of as-synthesized flower-like ZnO nanoparticles was evaluated from the MB degradation under UV irradiation. The ZnO nanopowders exhibit good degradation activities in an aqueous solution of MB, according to their size and morphology. The highest photocatalytic performance after 40 min of exposure to light (42%) was exhibited by ZnO nanoflowers synthesized without capping agent, while the addition of CTABr or *S. officinalis* extracts results in a decrease of degradation efficiency to 33% and 21%, respectively.

Antibacterial activity was tested against commonly used strains *E. coli*, *P. aeruginosa*, *S. aureus* and *C. albicans*. The minimum inhibitory concentrations of ZnO nanoflowers with plant extract were found in the range 10–20 µg/mL, larger than the values for the ZnO flowers without capping agents, but smaller compared to the ones of CTABr capped ZnO nanoparticles, respectively.

In summary, a novel microwave-assisted green synthesis method using *S. officinalis* extract as a capping agent is proposed as a low-cost, time and energy-efficient, environmentally friendly approach. The ZnO nanoflowers’ antibacterial and photocatalytic activities suggest that they can be used as industrial processes of dye removal and as an affordable non-toxic product in other applications based on antibacterial activity.

## Figures and Tables

**Table 1:** The synthesis conditions of ZnO samples prepared in the study.

Sample Code	Zn^2+^ Source	Reducing Agent	Structuring Agent
ZnO_1	Zn(NO_3_)_2_·6H_2_O	NaOH	-
ZnO_2	Zn(NO_3_)_2_·6H_2_O	NaOH	CTABr
ZnO_3	Zn(NO_3_)_2_·6H_2_O	NaOH	*S. officinalis* extract
ZnO_4	Zn(NO_3_)_2_·6H_2_O	*S. officinalis* extract	*S. officinalis* extract

**Table 2:** The size, size distribution and zeta potential of the ZnO samples prepared in the microwave irradiation using various capping and structuring agents.

Sample ^1^	Dm (nm)	PdI	Zeta Potential (mV)	Observations
ZnO_1	1214; >5500	0.363	−38.1	Large aggregates at >5500 nm.
ZnO_2	1068	0.218	−20.0	Monomodal distribution.
ZnO_3	863	0.295	−31.8	Monomodal distribution.
ZnO_4	42; 244	0.358	−16.2	Bimodal distribution with a major population at 42 nm and aggregates at 244 nm. Few larger aggregates.

^1^ ZnO_1 = sample synthesized in an alkaline medium without capping agent, ZnO_2 = synthesized in an alkaline medium in the presence of CTABr, ZnO_3 = synthesized in an alkaline medium in the presence of plant extract and ZnO_4 = synthesized only in the plant extract.

**Table 3:** Textural properties of the calcinated ZnO samples.

Sample	S_BET_ (m^2^·g^−1^)	S_BJH ads._ (m^2^·g^−1^)	S_BJH des._ (m^2^·g^−1^)	D_a_ (nm)	D_d_ (nm)	Total Pore Volume (cm^3^·g^−1^)
ZnO_1	2.292	3.69	7.005	3.237	3.301	0.0116
ZnO_2	8.355	8.021	15.001	3.540	3.835	0.0036
ZnO_3	3.257	3.011	4.710	4.294	3.294	0.0094

## Data Availability

Not applicable.

## Sample Availability

Samples of the compounds are available from the authors.
